# Supplementary material for: Test-retest reliability of regression dynamic causal modeling
Source: Netw Neurosci. 2022 Feb 1;6(1):135–60. doi: 10.1162/netn_a_00215 (PMC8959103; doi:10.1162/netn_a_00215)
Supplement: Supplementary file 1 [file netn-06-135-s001.pdf]

Frässle, S. & Stephan, K. E. (2022). Supporting information for "Test-retest reliability of regression dynamic causal modeling." *Network Neuroscience, 6*(1), 135–160. https://doi.org/10.1162/netn_a_00215

SUPPLEMENTARY MATERIAL

Test-retest reliability of regression dynamic causal modeling

Stefan Frässle, Klaas E. Stephan

SUPPLEMENTARY TABLES

Supplementary Table S1. Test-retest reliability of model parameter estimates for regression DCM and functional connectivity. Test-retest reliability of parameter estimates was assessed in terms of the intra-class correlation coefficient (ICC) between estimates of session 1 ("test") and session 2 ("retest") for a fixed (full) network architecture (i.e., classical rDCM and Pearson's correlation coefficient). Here, we report the mean (averaged across parameters) ICC value and 95% confidence interval (CI). Averaging of the connection-wise ICC values as well as computing the 95% CI was achieved by (i) transforming connection-wise ICC values to z-space using Fisher-z transformation, (ii) computing mean as well as lower and upper bound of the 95% CI in z-space, and finally (iii) back-transforming estimates to r-space. Test-retest reliability is reported for the connectivity and driving input parameters of rDCM (*middle*) as well as for the functional connectivity estimates (*right*). For both methods, results are shown for all HCP paradigms (i.e., rest, emotional processing, gambling, language, motor, relational processing, social cognition, and working memory) for the Schaefer atlas. Furthermore, results are shown for: ([a]) all parameters (*top row*), ([b]) significant parameters (*middle row*), and ([c]) top-1000 parameters (*bottom row*).

	**rDCM**		**FC**
	connectivity	*inputs*	
Fixed network			
	0.26 [-0.17 0.60][a]		0.16 [-0.25 0.52][a]
REST	0.32 [-0.11 0.65][b]	–	0.13 [-0.31 0.53][b]
	0.50 [-0.04 0.81][c]		0.20 [-0.41 0.69][c]
EMOTION	0.33 [-0.21 0.72]	0.07 [-0.40 0.51]	0.33 [-0.10 0.65]
	0.66 [0.11 0.90]	0.36 [0.36 0.36]	0.39 [-0.22 0.78]
	0.77 [0.49 0.90]	–	0.49 [-0.34 0.89]
GAMBLING	0.39 [-0.11 0.73]	0.33 [-0.12 0.66]	0.35 [-0.03 0.64]
	0.59 [0.18 0.83]	0.42 [0.03 0.70]	0.36 [-0.17 0.72]
	0.69 [0.37 0.86]	–	0.33 [-0.43 0.82]

LANGUAGE	0.42 [-0.09 0.76]	0.39 [-0.13 0.74]	0.37 [-0.07 0.69]
	0.46 [-0.08 0.79]	0.41 [0.21 0.58]	0.43 [-0.18 0.80]
	0.74 [0.41 0.90]	–	0.54 [-0.37 0.92]
MOTOR	0.32 [-0.21 0.71]	0.26 [-0.10 0.56]	0.35 [-0.04 0.64]
	0.54 [0.15 0.79]	0.39 [0.05 0.65]	0.38 [-0.17 0.75]
	0.63 [0.31 0.83]	–	0.43 [-0.33 0.85]
RELATIONAL	0.39 [-0.11 0.73]	0.42 [-0.13 0.77]	0.33 [-0.09 0.66]
	0.46 [-0.09 0.80]	0.56 [0.15 0.80]	0.37 [-0.24 0.77]
	0.71 [0.39 0.88]	–	0.45 [-0.38 0.88]
SOCIAL	0.42 [-0.09 0.75]	0.43 [-0.02 0.74]	0.36 [-0.07 0.68]
	0.46 [-0.09 0.79]	0.49 [0.13 0.74]	0.40 [-0.22 0.79]
	0.72 [0.41 0.88]	–	0.50 [-0.42 0.91]
WORKING MEMORY	0.33 [-0.23 0.72]	0.16 [-0.18 0.46]	0.34 [-0.03 0.64]
	0.58 [0.21 0.80]	0.27 [-0.08 0.57]	0.38 [-0.13 0.73]
	0.67 [0.34 0.86]	–	0.42 [-0.27 0.82]

Supplementary Table S2. Test-retest reliability of model parameter estimates for regression DCM and functional connectivity (sparsity constraints). Test-retest reliability of parameter estimates was assessed in terms of the intra-class correlation coefficient (ICC) between estimates of session 1 (“test”) and session 2 (“retest”) for sparsity constraints (i.e., rDCM with sparsity constraints and L1-regularized partial correlations). Here, we report the mean (averaged across parameters) ICC value and 95% confidence interval (CI). Averaging of the connection-wise ICC values as well as computing the 95% CI was done in z-space (see caption of Tab. 2 for details). Test-retest reliability is reported for the connectivity and driving input parameters of rDCM (*middle*) as well as for the functional connectivity estimates (*right*). For both methods, results are shown for all HCP paradigms (i.e., rest, emotional processing, gambling, language, motor, relational processing, social cognition, and working memory) for the Schaefer atlas. Furthermore, results are shown for: ([a]) all parameters (*top row*), ([b]) significant parameters (*middle row*), and ([c]) top-1000 parameters (*bottom row*).

	**rDCM**		**FC**
	connectivity	*inputs*	
Fixed network			
	0.03 [-0.27 0.32][a]		0.15 [-0.39 0.61][a]
REST	0.03 [-0.26 0.31][b]	–	0.52 [0.04 0.80][b]
	0.06 [-0.29 0.39][c]		0.57 [0.13 0.82][c]
EMOTION	0.25 [-0.24 0.64]	0.19 [-0.26 0.57]	0.05 [-0.41 0.49]
	0.46 [-0.16 0.82]	–	0.33 [-0.01 0.60]
	0.70 [0.36 0.87]	–	0.32 [-0.08 0.63]
GAMBLING	0.30 [-0.16 0.64]	0.27 [-0.19 0.63]	0.06 [-0.44 0.53]
	0.33 [-0.12 0.66]	0.41 [-0.03 0.71]	0.30 [-0.08 0.60]
	0.60 [0.22 0.82]	–	0.31 [-0.05 0.61]
LANGUAGE	0.29 [-0.15 0.63]	0.39 [-0.12 0.74]	0.09 [-0.44 0.57]
	0.31 [-0.09 0.63]	0.38 [0.15 0.57]	0.39 [-0.03 0.70]
	0.56 [0.11 0.82]	–	0.40 [-0.02 0.70]
MOTOR	0.35 [-0.10 0.68]	0.19 [-0.35 0.64]	0.07 [-0.39 0.50]
	0.43 [0.05 0.70]	–	0.33 [-0.05 0.63]
	0.49 [0.09 0.75]	–	0.35 [-0.05 0.65]
RELATIONAL	0.31 [-0.13 0.65]	0.41 [-0.13 0.76]	0.08 [-0.43 0.54]
	0.34 [-0.07 0.65]	0.53 [0.09 0.80]	0.35 [0.00 0.63]
	0.60 [0.19 0.83]	–	0.35 [-0.04 0.64]
SOCIAL	0.32 [-0.12 0.65]	0.38 [-0.08 0.70]	0.09 [-0.46 0.59]
	0.33 [-0.09 0.64]	0.45 [0.10 0.71]	0.39 [-0.01 0.69]
	0.59 [0.16 0.83]	–	0.42 [0.01 0.71]
WORKING MEMORY	0.27 [-0.14 0.60]	0.12 [-0.22 0.44]	0.08 [-0.38 0.50]
	0.27 [-0.08 0.56]	–	0.35 [-0.04 0.64]
	0.30 [-0.10 0.62]	–	0.36 [-0.02 0.65]

SUPPLEMENTARY FIGURES

Supplementary Figure S1

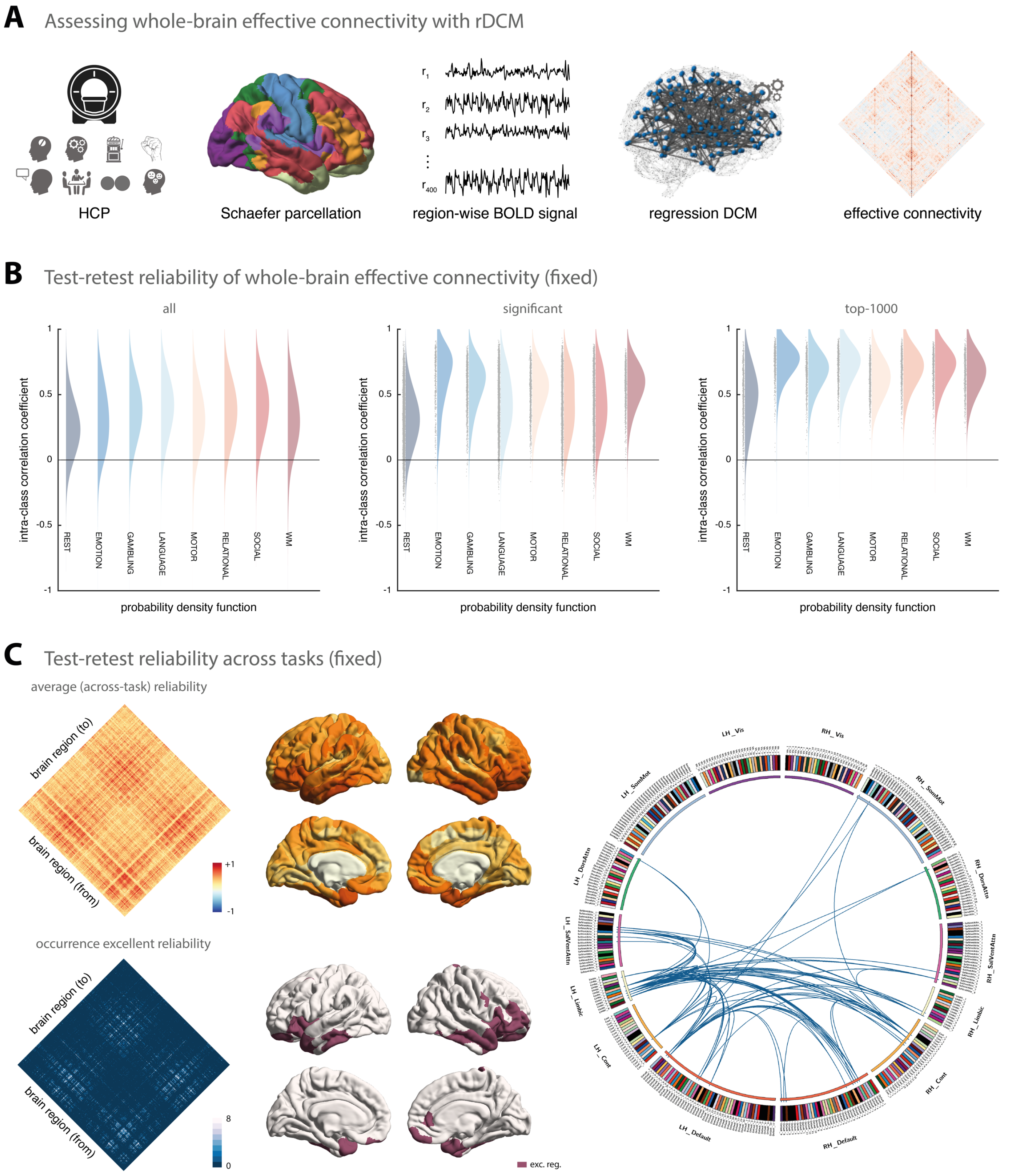

Supplementary Figure S1: Test-retest reliability of regression DCM for a fixed network architecture. (A) Methodological overview. Resting-state and task-based fMRI data from the Human Connectome Project (HCP) is used for the analysis. Region-wise BOLD signal time series were extracted from a whole-brain parcellation scheme (e.g., the Glasser atlas) and effective connectivity was inferred at the whole-brain level using regression dynamic causal modeling (rDCM). The rDCM parameter estimates were then subjected to group-level consistency and test-

retest reliability analyses. **(B)** Estimates of the probability density functions (using the nonparametric kernel smoothing of *fitdist.m* implemented in MATLAB) of the connection-wise intra-class correlation coefficient (ICC) for the resting-state and all 7 tasks (i.e., emotional processing, gambling, language, motor, relational processing, social cognition, and working memory) for the Schaefer atlas. Results are shown when considering all connections (*left*), significant connections (*middle*), and the top-1000 connections (*right*). **(C)** Mean (averaged across all paradigms) test-retest reliability (*top, left*) as well as how often (i.e., in how many paradigms) a connection showed excellent reliability (*bottom, left*). Mean test-retest reliability projected onto the cortical surface (*top, middle*) and the cortical location of all regions that are linked via connections that show excellent reliability in all 8 paradigms (*bottom, middle*). Connectogram showing the connections with excellent reliability in all 8 paradigms (*right*). The connectogram was produced using Circos (publicly available at http://circos.ca/software/). Parcels of the Schaefer atlas can be assigned to the resting state networks (RSN) defined in Yeo et al. (2011), which include visual (Vis), somatosensory-motor (SomMot), dorsal attention (DorsAttn), ventral attention (SalVentAttn), limbic (Limbic), control (Cont), and default mode network (Default).

Supplementary Figure S2

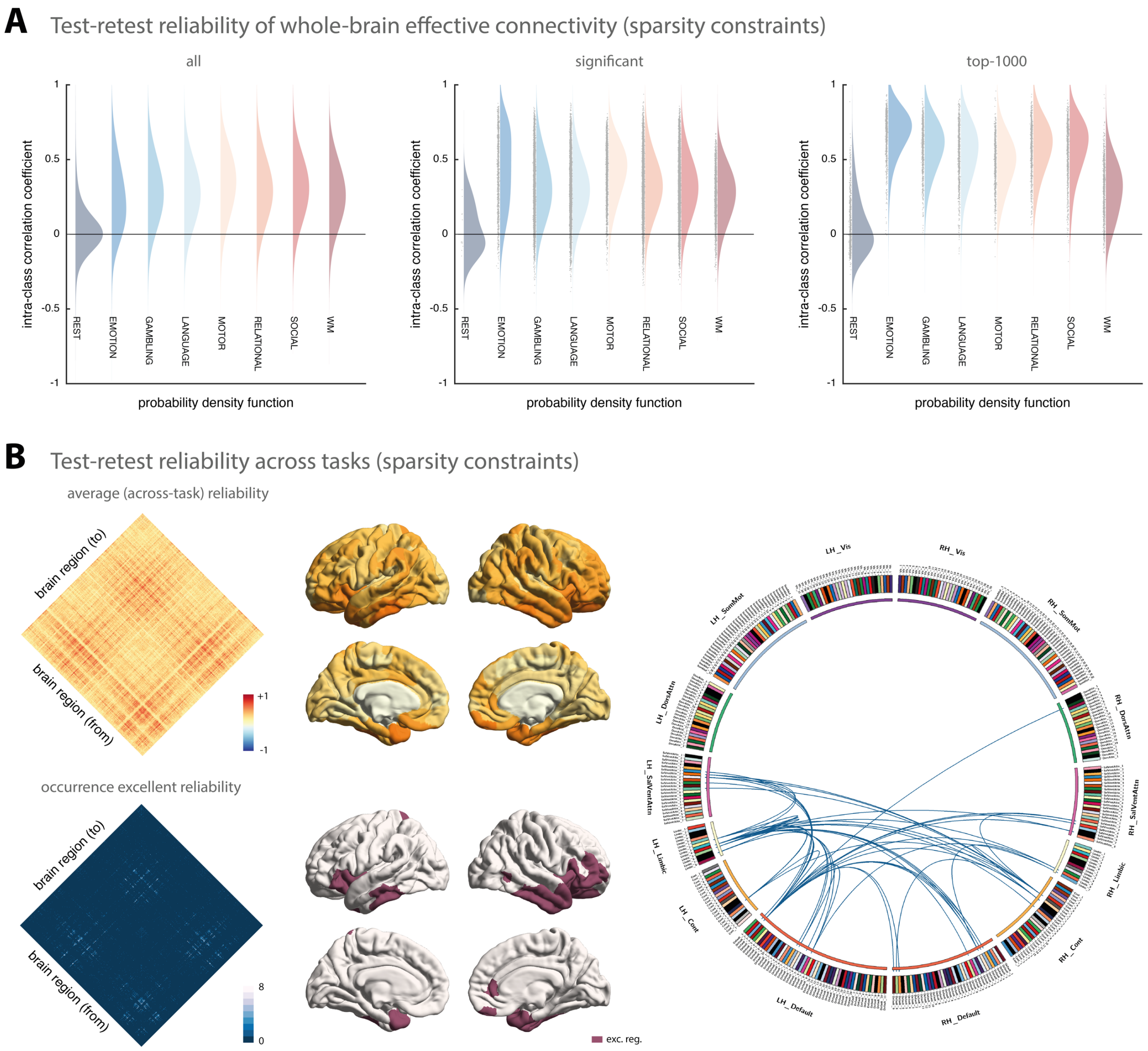

Supplementary Figure S2: Test-retest reliability of regression DCM with sparsity constraints. (A) Estimates of the probability density functions (using the nonparametric kernel smoothing of *fitdist.m* implemented in MATLAB) of the connection-wise intra-class correlation coefficient (ICC) for the resting-state and all 7 tasks (i.e., emotional processing, gambling, language, motor, relational processing, social cognition, and working memory) for the Schaefer atlas. Results are shown when considering all connections (*left*), significant connections (*middle*), and the top-1000 connections (*right*). **(B)** Mean (averaged across all paradigms) test-retest reliability (*top, left*) as well as how often (i.e., in how many paradigms) a connection showed excellent reliability (*bottom, left*). Mean test-retest reliability projected onto the cortical surface (*top, middle*) and the cortical location of all regions that are linked via connections that show excellent reliability in at least 6 paradigms (*bottom, middle*). Connectogram showing the connections with excellent reliability in at least 6 paradigms (*right*). The connectogram was produced using Circos (publicly available at http://circos.ca/software/). Parcels of the Schaefer atlas can be assigned to the

resting state networks (RSN) defined in Yeo et al. (2011), which include visual (Vis), somatosensory-motor (SomMot), dorsal attention (DorsAttn), ventral attention (SalVentAttn), limbic (Limbic), control (Cont), and default mode network (Default).

Supplementary Figure S3

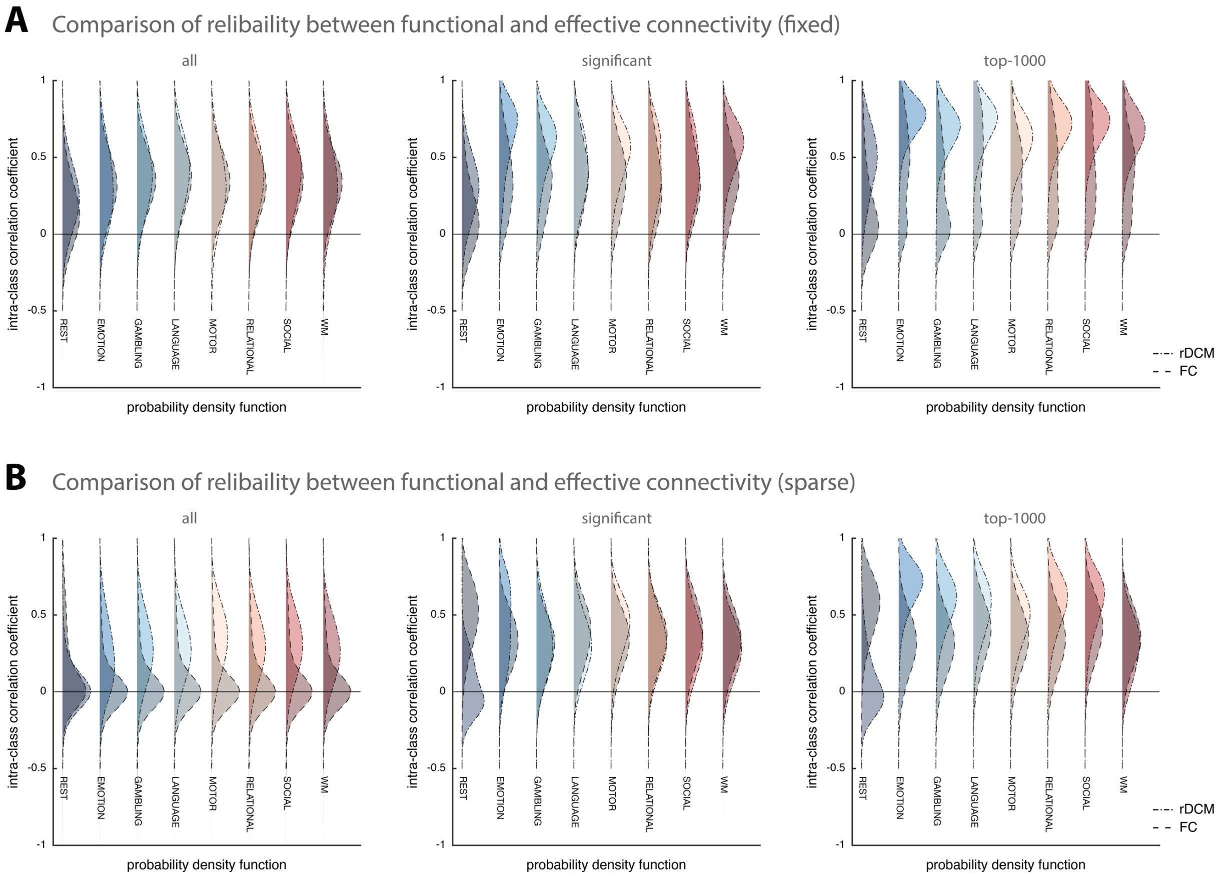

Supplementary Figure S3: Comparison of test-retest reliability between regression DCM and functional connectivity. (A) Estimates of the probability density functions (using the nonparametric kernel smoothing of *fitdist.m* implemented in MATLAB) of the connection-wise intra-class correlation coefficient (ICC) for the resting-state and all 7 tasks (i.e., emotional processing, gambling, language, motor, relational processing, social cognition, and working memory) for the Schaefer atlas for fixed (full) connectivity methods (i.e., classical rDCM and Pearson's correlation coefficient), and **(B)** sparse connectivity methods (i.e., rDCM with sparsity constraints and L1-regularized partial correlations). Probability density functions representing rDCM results are shown with dot-dashed lines and lighter colors, whereas probability density functions representing functional connectivity results are shown with dashed lines and darker colors. For each connectivity variant, results are shown when considering all connections (*left*), significant connections (*middle*), and the top-1000 connections (*right*).

References

Yeo, B.T., Krienen, F.M., Sepulcre, J., Sabuncu, M.R., Lashkari, D., Hollinshead, M., Roffman, J.L., Smoller, J.W., Zollei, L., Polimeni, J.R., Fischl, B., Liu, H., Buckner, R.L., 2011. The organization of the human cerebral cortex estimated by intrinsic functional connectivity. J Neurophysiol 106, 1125-1165.
